# Exosome-dependent and independent mechanisms are involved in prion-like transmission of propagated Cu/Zn superoxide dismutase misfolding

**DOI:** 10.4161/19336896.2014.983398

**Published:** 2014-12-31

**Authors:** Leslie I Grad, Edward Pokrishevsky, Judith M Silverman, Neil R Cashman

**Affiliations:** Department of Medicine (Neurology), Brain Research Center; University of British Columbia; Vancouver, BC Canada

**Keywords:** amyotrophic lateral sclerosis, exosome, intercellular transmission, protein misfolding, prion-like, superoxide dismutase

## Abstract

Amyotrophic lateral sclerosis (ALS), a fatal adult-onset degenerative neuromuscular disorder with a poorly defined etiology, progresses in an orderly spatiotemporal manner from one or more foci within the nervous system, reminiscent of prion disease pathology. We have previously shown that misfolded mutant Cu/Zn superoxide dismutase (SOD1), mutation of which is associated with a subset of ALS cases, can induce endogenous wild-type SOD1 misfolding in the intracellular environment in a templating fashion similar to that of misfolded prion protein. Our recent observations further extend the prion paradigm of pathological SOD1 to help explain the intercellular transmission of disease along the neuroaxis. It has been shown that both mutant and misfolded wild-type SOD1 can traverse cell-to-cell either as protein aggregates that are released from dying cells and taken up by neighboring cells via macropinocytosis, or released to the extracellular environment on the surface of exosomes secreted from living cells. Furthermore, once propagation of misfolded wild-type SOD1 has been initiated in human cell culture, it continues over multiple passages of transfer and cell growth. Propagation and transmission of misfolded wild-type SOD1 is therefore a potential mechanism in the systematic progression of ALS pathology.

## ALS is A Prion-like Neuromuscular Proteinopathy

Amyotrophic lateral sclerosis (ALS) is a rapidly progressive fatal neuromuscular disorder characterized by degeneration of the upper and lower motor neurons causing progressive paralysis and spasticity that affects the muscles of mobility, speech, swallowing and respiration.^1^ Half of affected individuals die within 3 years, and less than 20% survive for more than 5 years.^2^ The etiology of ALS is unknown; however, similar to other neurodegenerative diseases such as Alzheimer and Creutzfeldt-Jakob diseases, the disease can be divided into 2 categories, sporadic and familial. Approximately 90% of ALS cases are sporadic (SALS) where some predisposing gene mutations have been identified, or with no known genetic components. The remainder of cases are familial (FALS),^3^ which are predominantly associated with Mendelian-inherited mutations in genes encoding the Cu/Zn superoxide dismutase (SOD1), TAR-DNA binding protein 43 (TDP-43), fused in sarcoma/translocated in liposarcoma (FUS/TLS), and C9ORF72.^4,5^ Clinically, SALS and FALS have similar presentations suggesting that a common downstream pathogenic mechanism may pertain, regardless of disease origin. ALS is categorized as a proteinopathy; spinal cord histology of ALS patients often reveals abnormal accumulations of ubiquitinated proteinaceous inclusions in motor neurons and neural accessory cells. These large aggregates are thought to be the result of the aggregation of non-natively folded proteins, which can be triggered to misfold by a multitude of factors, including mutation, oxidation, aberrant post-translational modification, dysfunctional proteostasis and prion-like protein propagation.

Prion-like mechanisms have been proposed and identified for certain proteins involved in ALS, including SOD1, a small soluble and ubiquitously-expressed free radical scavenging enzyme that normally exists as a protease-resistant homodimer.^6^ In its native state SOD1 is a highly stable protein, however, it is prone to destabilization and subsequent aggregation when aberrantly oxidized or mutated. Recent experimental evidence supports that a protein-to-protein conversion process observed for both mutant and over-expressed wild-type SOD1 (WTSOD1) can be self-sustaining.^7^ Induced misfolding of SOD1 can persist even in the absence of the original misfolded ‘seed’ in cell culture^7,8^, suggesting that newly misfolded SOD1 can act as template for subsequent cycles of misfolding. In addition, applying exogenously-aggregated mutant human SOD1 can induce subsequent aggregation of soluble transgenically-expressed mutant human SOD1 in mouse neuroblastoma cells.^8^ The observation that WTSOD1 is capable of facilitating intracellular propagated SOD1 misfolding further implicates its misfolding as a common pathogenic mechanism of ALS regardless of disease etiology. Interestingly, the prion-like paradigm of propagated SOD1 misfolding may explain the clinical observations of ALS disease progression, which suggests that motor neuron injury begins at one, or possibly more,^9^ focal points followed by progressive and contiguous spread of disease through the neuroaxis, *i.e.* pathology appears to spread without skipping neuroanatomical regions.^10^ It should be noted that prion-like behavior in SOD1 has yet to be directly observed and confirmed *in vivo*; the experimental evidence generated to date is generally restricted to cellular models and indirect observations in ALS mouse models that may or may not reflect the mechanism occurring in ALS patients.

## Intercellular Transmission of Propagated WTSOD1 Misfolding

Intermolecular conversion of native WTSOD1 to a misfolded conformer has been demonstrated within living cells.^7^ Introduction of a misfolded mutant form of SOD1 into human cells imparts its misfold onto natively-folded WTSOD1, as detected by conformation-specific antibodies^7,11^ whose epitopes are only accessible when SOD1 is misfolded. More importantly, misfolded WTSOD1 persists over several weeks in cell culture, long after the exogenous mutant SOD1 has dissipated through plasmid depletion and protein degradation, strongly suggesting that misfolded WTSOD1 self-propagates intracellularly. We also detect misfolded WTSOD1 in cell culture medium from mutant SOD1-transfected human cells. This finding indicated that cells containing mutant-induced misfolded human WTSOD1 might have the capacity to transmit misfolded SOD1 from cell to cell, facilitating the spread of propagated misfolding in a self-sustaining manner. Indeed, when conditioned medium collected from mutant SOD1-transfected human cells was placed on untransfected cells, the endogenous WTSOD1 underwent misfolding. The induction is sensitive to siRNA-mediated down-regulation of endogenous SOD1 in treated cells, confirming that resident WTSOD1 in recipient cells is required as substrate for induced protein misfolding by exogenous misfolded seed and rules out simple uptake of misfolded SOD1 from the medium as an explanation for the presence of misfolded WTSOD1 in untransfected cells. Consistent with prion-like characteristics, SOD1 misfolding can be serially passaged in cell cultures via sequential conditioned media incubation. This is further testament to the ability of misfolded WTSOD1 to perpetually self-sustain through different populations of cells. Analysis of the conditioned media by ultracentrifugation reveals that the transmission particle is relatively dense, consistent with large protein aggregates and extracellular vesicles. Regardless of the nature of the transmission particle, misfolded WTSOD1 is directly exposed to the extracellular milieu as conditioned media incubated with misfolded SOD1-specific antibodies prior to its addition to untransfected cells nearly abolishes propagated WTSOD1 misfolding.

Mechanistically, misfolded WTSOD1 behaves as a prion-like protein on a molecular level *in vitro*. The logical next question is whether or not this mechanistic finding is relevant to human disease. Misfolded and aggregated SOD1 is invariably detected in post-mortem spinal cord histology of ALS patients with mutations in the gene encoding SOD1. However, historically there is less certainty regarding the presence of aggregated SOD1 in ALS patients lacking any mutation in the *SOD1* gene. More recently, a collection of biochemical, genetic and immunological evidence of misfolded SOD1 in cases of SOD1-excluded sporadic ALS ^12-16^ supports the hypothesis that misfolded WTSOD1 plays a pathological role in the disease, although this hypothesis is not one of consensus.^17^ Using our misfolded SOD1-specific antibodies, we conducted a large immunohistochemical analysis of post-mortem spinal cord tissue from control (N = 5), sporadic (N = 20) and familial (N = 8; 5 with mutated SOD1) ALS cases. Regardless of the presence of a mutation in the *SOD1* gene, all ALS samples showed the presence of misfolded SOD1; staining was not detected in controls. We observed that the size, number and location of SOD1 aggregates differed significantly between SOD1-FALS cases and non-SOD1 FALS and SALS cases. For example, neurons from mutant SOD1-linked FALS patient spinal cord stained for numerous cytoplasmic inclusions that were relatively rare in cases where mutations in SOD1 were excluded. However, quantitative immunoprecipitation showed comparable levels of misfolded SOD1 between the different classes of ALS patients, suggesting that misfolded WTSOD1 in sporadic ALS patients may take the form of smaller soluble oligomers rather than large proteinaceous inclusions. Given the elevated cytotoxicity of misfolded human WTSOD1 observed in motor neuron-like cells,^16^ its propensity for propagated protein misfolding, efficient intercellular transfer and presence in ALS patient pathology, there is strong evidence to support a key pathogenic role for misfolded WTSOD1 in human disease.

## Mechanisms of Intercellular Transmission of Misfolded SOD1

**Figure 1.**
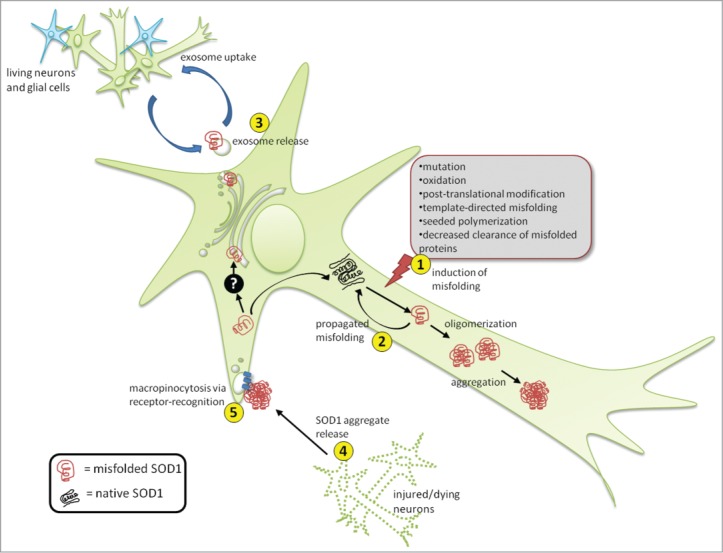
Propagation and transmission of misfolded SOD1. SOD1 misfolding can be induced by a variety of intracellular and extracellular stresses (1). Once a misfolded template is present, it can induce subsequent cycles of template-directed misfolding, converting neighboring native SOD1 molecules into pathological isoforms, which can subsequently form oligomers and aggregates over time (2). Presumably during the early stages of disease, misfolded SOD1 can also accumulate in the ER-Golgi system by an unknown mechanism (black box), where it can enter the vesicle-mediated secretory pathway, becoming selectively incorporated onto the outer surface of exosomes, exit the cell via secretion (3) and be taken up by neighboring cells. Alternatively, during later stages of disease when neural cells are injured and dying, large proteinaceous aggregates containing misfolded SOD1 are released (4) and subsequently taken up by neighboring cells via macropinocytosis (5). Uptake through this mechanism appears specific making the presence of certain cell surface receptors mediating uptake likely.

The mechanisms responsible for the intercellular transmission of propagated SOD1 misfolding are not fully elucidated. Extracellular transport vesicles have been suggested as a possible mechanism for the progression of neurodegenerative disease pathology, especially between living neural cells. One class of vesicle in particular, called exosomes, have been implicated in the pathogenesis of the prion diseases.^18^ Similarly, exosomes were previously established as a secretory mechanism for both wild-type and mutant SOD1 in motor neuron-like cells.^19,20^ Our analysis shows that misfolded human WTSOD1 can be released from mouse motor neuron-like cells on exosomes and is subsequently taken up by neighboring cells. Interestingly, we find that misfolded SOD1 is localized to the outer surface of the exosomal membrane^16^, as opposed to native SOD1, which normally resides in the exosomal lumen.^21^ The surface localization of misfolded SOD1 allows for its recognition and subsequent deactivation by potential pharmacological and immunological therapeutics. As an alternative mechanism of transmission, aggregates of mutant^8^ and misfolded WTSOD1^16^ can be released from dying cells and efficiently taken up by neighboring cells via the process of macropinocytosis. Together our findings suggest that uptake of misfolded WTSOD1 can occur through both exosome-dependent and independent means (**Fig. 1**). Exosome-independent uptake of SOD1 is not aggregate-specific as aggregated forms are taken up as efficiently as non-aggregated forms. However, the process does show specificity, as an irrelevant cellular aggregate, such as glutathione S-transferase, is not taken up in the same manner as SOD1, suggesting the involvement of receptors in this process (**Fig. 1**). Previous work with mouse microglial cells found that scavenger receptors specifically participate in the uptake of aggregated SOD1 ^22^, whereas heparin sulfate proteoglycans have been shown to be involved in aggregate uptake of tau, α-synuclein, and prion protein^23,24^ in other neurodegenerative diseases. Given the specificity of this pathway, as well as the localization of misfolded SOD1 to the surface of exosomes, other as yet identified factors are at play that govern the intracellular trafficking and extracellular survival of misfolded SOD1 in ALS.

## Misfolded SOD1 Plays by Its Own Rules

**Figure 2.**
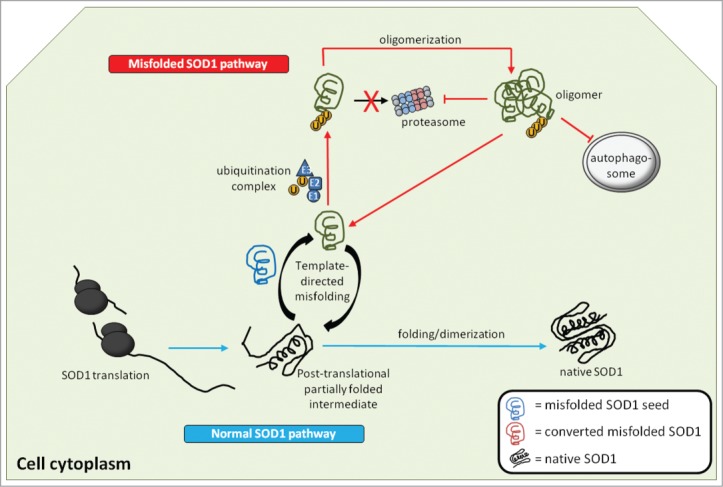
Generation and actions of misfolded SOD1 inside the cell. Native WTSOD1 or near-native SOD1 mutants are often highly protease resistant making their ability to misfold thermodynamically unfavourable. One explanation is that misfolded SOD1 seed (blue) utilizes post-translational intermediates of WT or mutant that remain partially unfolded and susceptible to induced pathological misfolding. Once populations of misfolded SOD1 (green) accumulate and begin to oligomerize, they become difficult to degrade via the ubiquitin-proteasome system, despite being conjugated with ubiquitin. It is also possible that misfolded SOD1 itself can act as an inhibitor of both the proteasome and autophagic systems, through as yet identified mechanisms, thereby allowing for aberrantly folded protein to elude proteostasis and build up within the cell.

Although our experimental evidence supports prion-like propagation and transmission of misfolded SOD1, it remains poorly understood how misfolded SOD1 avoids degradation by the proteostatic machinery of the cell, incorporates itself into the secretory pathway such that it remains associated with the surface of extracellular vesicles, and what specific receptors, if any, control the uptake of misfolded WTSOD1 presented on exosomes or vesicle-free aggregates. Along with other pathological proteins, misfolded SOD1 is targeted for degradation through ubiquitination^25^, but can escape this process through oligomerization and aggregation (**Fig. 2**). Larger multimeric forms of protein are known to become more thermodynamically stable and thus less sensitive to protease recognition and degradation.^26^ Misfolded mutant SOD1 has been shown to have toxic effects on both autophagy and the proteasome-dependent degradation.^27,28^ Reduced proteasome subunit levels and activity are a common molecular signature in both FALS and SALS patients,^29^ suggesting deficits in proteasome activity is part of the molecular disease pathology in humans, and is independent of mutations in the *SOD1* gene. Mutations in several genes encoding proteostatic proteins, such as ubiquilin 2^30^ or p62^31^ cause ALS; however, it remains to be seen what triggers proteostatic dysfunction in the absence of direct mutation within the proteostatic machinery.

Lack of degradation of misfolded SOD1 inside the cell is only the beginning of a protein misfolding cascade in which the mechanism for incorporation into the vesicle-mediated secretory pathway has yet to be elucidated. The presence of misfolded mutant SOD1 often compromises the secretory pathway.^32^ Moreover, mutant SOD1 oligomers accumulate in the endoplasmic reticulum-Golgi compartments as a precursor to its secretion^33^ (**Fig. 1**), a trafficking step often reserved for cargo heading for degradation to the lysosome or secretion directly into the extracellular space by late endosomes. Such a route is not routinely taken by a normally soluble cytosolic protein. Taken together, it is clear that SOD1 in its pathological form can hijack the vesicle-mediated secretory system to ensure its survival not only within the cell, but also its transmission to other cells in order to perpetuate new rounds of toxic protein misfolding. One can speculate that the aberrant conformation generated by misfolded SOD1 presents a trafficking signal not normally accessible in the native state that allows it to skirt the proteostatic system, while simultaneously being incorporated into the secretory system of the cell, making it a true rogue entity within the cell.

Finally, the conformational state of the recruited SOD1 species in the propagated protein misfolding process – whether natively folded or partially unfolded– remains to be determined. It seems paradoxical that WTSOD1, or even an enzymatically functional mutant SOD1 in a near-native state, which is highly thermodynamically stable can be so readily recruited into a pathologically misfolded form. FALS-linked mutant SOD1 species are known to have delayed post-translational folding kinetics, thereby increasing the population of partially folded intermediates.^34^ These partially-folded protein intermediates could provide an ideal substrate for recruitment (**Fig. 2**). However, the same cannot easily explain the recruitment of WTSOD1, which possesses relatively quicker folding kinetics compared to mutant SOD1, producing significantly smaller populations of partially-folded intermediates. It is possible other mechanisms are at work in the ALS disease state that slow post-translational folding of WTSOD1, making it more susceptible for recruitment to misfold into a pathological misfolded form when it is recognized by misfolded mutant or WTSOD1 template; however, such factors have yet to be identified. Nonetheless, a solid foundation supporting prion-like propagated misfolding for SOD1 has been established, which utilizes both secretory vesicles and vesicle-free aggregates for its intercellular transmission. Furthermore, WTSOD1 can be efficiently converted into a misfolded conformer that is itself propagation- and transmission-competent in living human cells. That, combined with the evidence that misfolded WTSOD1 is present in mutant SOD1-excluded cases of ALS, suggests a key role for WTSOD1 in ALS disease pathology.
